# Genome Sequence of Airborne *Acinetobacter* sp. Strain 5-2Ac02 in the Hospital Environment, Close to the Species of *Acinetobacter towneri*

**DOI:** 10.1128/genomeA.01343-16

**Published:** 2016-12-08

**Authors:** Beathriz G. V. Barbosa, Laura Fernandez-García, Eva Gato, Maria López, Lucia Blasco, Robson Souza Leão, Rodolpho M. Albano, Begoña Fernández, Felipe-Fernández Cuenca, Álvaro Pascual, German Bou, Elizabeth A. Marques, María Tomás

**Affiliations:** a Departamento de Microbiologia, Inmunologia e Parasitologia-FCM/UERJ, Rio de Janeiro, Brazil; b Departamento de Microbiologia, Complejo Hospitalario Universitario, A Coruña, INIBIC (Sergas)-UDC, La Coruña, Spain; c Departamento de Bioquímica, Instituto de Biologia Roberto Alcântara Gomes, Universidade do Estado do Rio de Janeiro, Rio de Janeiro, Brazil; d Unidad Intercentros de Enfermedades Infecciosas, Microbiología y Medicina Preventiva, Hospital Universitario Virgen Macarena, Seville, Spain; e Spanish Network for Research in Infectious Diseases (REIPI RD12/0015), Seville, Spain; f Biomedical Institute of Seville (IBiS), Seville, Spain

## Abstract

*Acinetobacter* spp. are found in 53% of air colonization samples from the hospital environment. In this work, we sequenced all the genome of airborne *Acinetobacter* sp. strain 5-2Ac02. We found important features at the genomic level in regards to the rhizome. By phylogenetic analysis, *A. towneri* was the species most closely related to *Acinetobacter* sp. 5-2Ac02.

## GENOME ANNOUNCEMENT

*Acinetobacter* species can be found in any environment: water, soil, body surface, etc., and is one of the most dangerous nosocomial pathogens in the world (1), being found principally in intensive care units (ICUs) (2). In the last few years, it was discovered that members of the genus *Acinetobacter* can be transmitted by air (3), although it is true that air contamination when there are any patients infected is rare. However, some researchers refer to intense *Acinetobacter* species air contamination when there are infected patients around (4, 5). Nowadays, little is known about this method of transmission; therefore, many researchers are studying this transmission method.

In this study, we report the genome sequence of airborne *Acinetobacter* sp. strain 5-2Ac02, which was collected from the air in an ICU of a hospital in Rio de Janeiro, Brazil. Genomic DNA was isolated using the Wizard genomic DNA kit (Promega). Genome sequencing was determined using an Ilumina MiSeq system. The final draft genome was annotated using the RAST server to identify the protein-coding genes, rRNA, and tRNA genes, and to assign functions to these genes. The predicted open reading frames (ORFs) were confirmed using BlastP in the Protein Data Bank (PDB) and COG databases from NCBI and InterProScan. Using the Antibiotic Resistance Database, we could predict the antibiotic resistance genes. The insertion sequences (IS) were analyzed by IS Finder software. Antimicrobial susceptibility testing was performed according to CLSI methods (2014), and disinfectant susceptibility testing was performed by microdilution. Phylogenetic analysis was carried out by average nucleotide identity (ANI), single nucleotide polymorphism analysis (snpTree), and RNA polymerase beta subunit sequence (*rpoB*) analysis. A matrix-assisted laser desorption ionization–time of flight mass spectrometry (MALDI-TOF MS) dendrogram was generated (Bruker Daltonics).

The genome includes a chromosome of 2,951,447 bp, with a 40.9% G+C content. The functional annotation of the circular chromosome showed a total of 2,795 predicted coding sequences (CDSs) and suggests that 1,281 (46%) of these CDSs could be assigned a biological function included in 380 subsystems (RAST software). There were a total of 97 RNAs genes identified. We studied the rhizome of this genome (6). In the analysis of the resistome, we found the presence of TerC family proteins from the *ter* operon (*terZABCDEF)*; we also found *klaA* and *klaB* genes from the *kil* operon, which is in association with the previous one, as said by O’Gara et al. (7). One arsenic operon, arsC1-arsR-arsC2-ACR3-arsH was studied. This operon organization has only been described in the *Pseudomona stutzeri* TS44 (8). Moreover, this strain showed an MIC to arsenic of >2,048 mg/liter. Finally, an ISAba*3*-like transposase was present upstream of *bla*_OXA-58_, which determines its silence state (MICs to imipenem and meropenem, 0.06 and 0.03 mg/liter, respectively) (9). For the persistome, we found four different toxin-antitoxin systems: RelEB (4 gene cassettes), ParDE, HipBA, and HigBA, with all of them related to persistence, among other things (10). For the mobilome, the percentage of insertion sequences (ISs) in this strain was about 5%, which is higher than that of clinical *Acinetobacter* species. Finally, posterior phylogenetic analysis suggested that *Acinetobacter* sp. 5-2Ac02 is a new species of *Acinetobacter*, with *A. towneri* eing the most closely related one (94.39 by ANI score, 90% by *rpoB* score gene, and 2.127 MALDI-TOF MS score).

### Accession number(s).

This whole-genome shotgun project has been deposited at DDBJ/ENA/GenBank under the accession no. MKQS00000000. The version described in this paper is version MKQS01000000 (BioProject PRJNA345289).
